# Pooled testing conserves SARS-CoV-2 laboratory resources and improves test turn-around time: experience on the Kenyan Coast

**DOI:** 10.12688/wellcomeopenres.16113.2

**Published:** 2021-02-03

**Authors:** Charles N. Agoti, Martin Mutunga, Arnold W. Lambisia, Domtila Kimani, Robinson Cheruiyot, Patience Kiyuka, Clement Lewa, Elijah Gicheru, Metrine Tendwa, Khadija Said Mohammed, Victor Osoti, Johnstone Makale, Brian Tawa, Calleb Odundo, Wesley Cheruiyot, Wilfred Nyamu, Wilson Gumbi, Jedidah Mwacharo, Lydia Nyamako, Edward Otieno, David Amadi, Janet Thoya, Angela Karani, Daisy Mugo, Jennifer Musyoki, Horace Gumba, Salim Mwarumba, Bonface M. Gichuki, Susan Njuguna, Debra Riako, Shadrack Mutua, John N. Gitonga, Yiakon Sein, Brian Bartilol, Shaban J. Mwangi, Donwilliams O. Omuoyo, John M. Morobe, Zaydah R. de Laurent, Philip Bejon, Lynette Isabella Ochola-Oyier, Benjamin Tsofa

**Affiliations:** 1 Kenya Medical Research Institute-Wellcome Trust Research Programme, Centre for Geographic Medicine Research, Kilifi, Kenya; 2 Department of Biomedical Sciences, Pwani University, Kilifi, Kenya; 3 Nuffield Department of Medicine, Centre for Clinical Vaccinology and Tropical Medicine, Churchill Hospital, University of Oxford, Oxford, UK

**Keywords:** COVID-19, Pooled testing, SARS-CoV-2, Kilifi, Kenya

## Abstract

**Background.** International recommendations for the control of the coronavirus disease 2019 (COVID-19) pandemic emphasize the central role of laboratory testing for severe acute respiratory syndrome coronavirus 2 (SARS-CoV-2), the etiological agent, at scale. The availability of testing reagents, laboratory equipment and qualified staff are important bottlenecks to achieving this. Elsewhere, pooled testing (i.e. combining multiple samples in the same reaction) has been suggested to increase testing capacities in the pandemic period.

**Methods.** We discuss our experience with SARS-CoV-2 pooled testing using real-time reverse transcription polymerase chain reaction (RT-PCR) on the Kenyan Coast.

**Results.** In mid-May, 2020, our RT-PCR testing capacity for SARS-CoV-2 was improved by ~100% as a result of adoption of a six-sample pooled testing strategy. This was accompanied with a concomitant saving of ~50% of SARS-CoV-2 laboratory test kits at both the RNA extraction and RT-PCR stages. However, pooled testing came with a slight decline of test sensitivity. The RT-PCR cycle threshold value (ΔCt) was ~1.59 higher for samples tested in pools compared to samples tested singly.

**Conclusions.** Pooled testing is a useful strategy to increase SARS-CoV-2 laboratory testing capacity especially in low-income settings.

## Introduction

**Figure 1.**
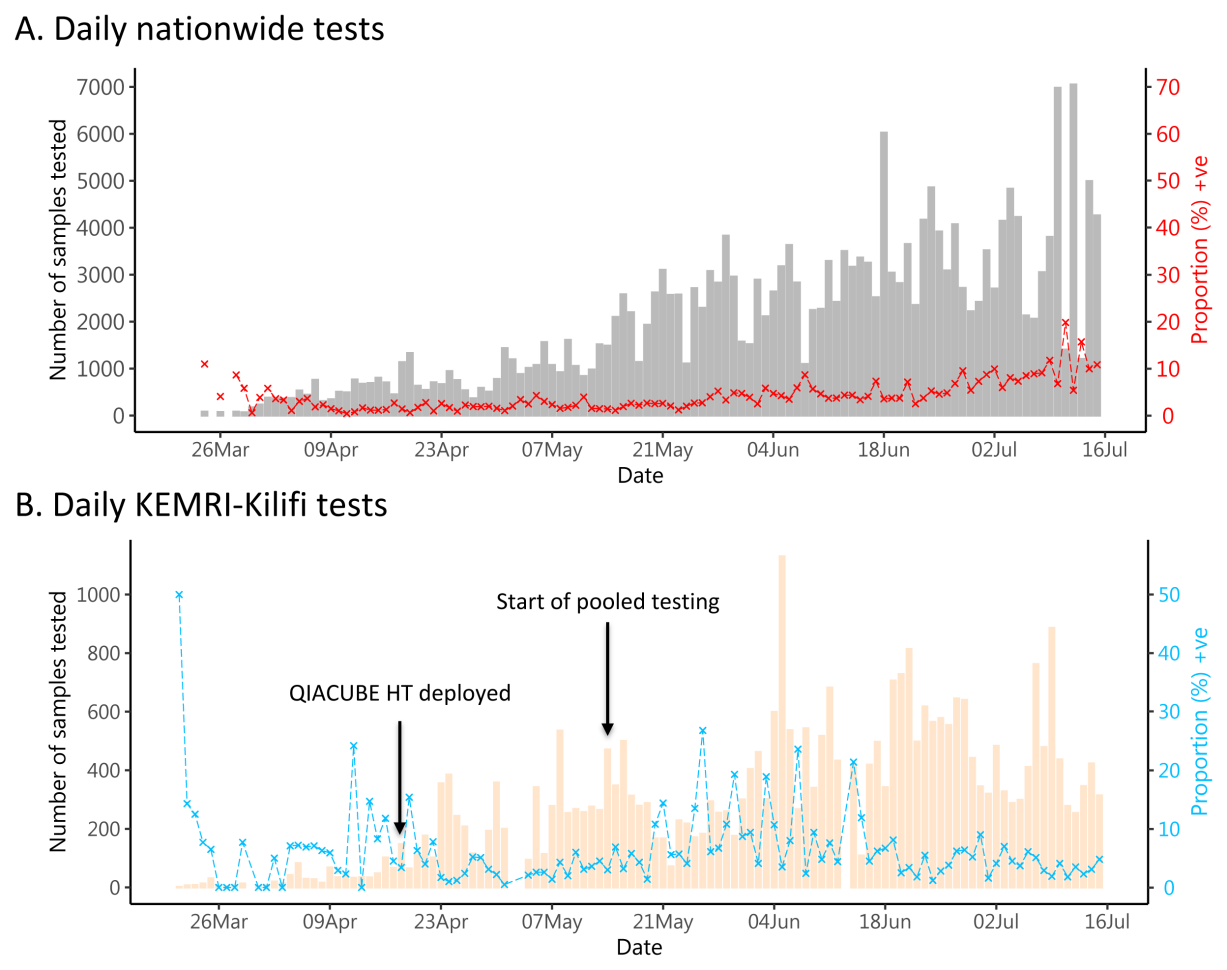
Severe acute respiratory syndrome coronavirus 2 (SARS-CoV-2) laboratory testing in Kenya between 21 ^th^ March and 15 ^th^ July 2020. Panel **A**, the bars show reported daily nationwide tests. Panel **B**, bars show the daily tests undertaken at the KEMRI-Kilifi laboratory and when major protocol changes were implemented. In both panels the secondary y-axis shows the daily proportion of tests positive indicated by the dashed line.

In Kenya, the first case of severe acute respiratory syndrome coronavirus 2 (SARS-CoV-2) infection, the etiological agent of coronavirus disease 2019 (COVID-19), was confirmed on the 12 ^th^ March 2020 ^1^. Since then the number of confirmed cases has risen steadily, each day, and as of 15 ^th^ July 2020, a total of 11,252 SARS-CoV-2 positives had been confirmed in the country from 225,495 samples tested, ~5.0% positivity rate overall ^2^. Scaling up of testing to enhance early case detection, isolation, treatment and to guide contact tracing has been a cornerstone strategy, worldwide, in managing the COVID-19 pandemic ^3^. Between 15 ^th^ May and 15 ^th^ July 2020, an average 3,046 laboratory tests were performed daily in Kenya ( Figure 1, panel A). Increasing the number of daily tests is a challenge for local laboratory capacity.

Real-time reverse transcription polymerase chain reaction (RT-PCR) is the gold standard method for SARS-CoV-2 diagnosis ^4^. The diagnostic process is initiated by viral nucleic acid purification from a suspected patient sample, followed by concurrent target nucleic acid amplification and detection. Soon after 30 ^th^ January 2020, when COVID-19 was declared a public health emergency of international concern, SARS-CoV-2 diagnostics were recognized as an important bottleneck in the efforts to effectively contain the epidemic ^5^. Laboratory testing capacity may be limited by the unavailability of equipment, reagents and qualified staff. As a result, more efficient testing protocols have since been pursued to facilitate the mantra “test, trace, isolate and treat”. One such protocol is pooled testing ^5, 6^.

**Figure 2.**
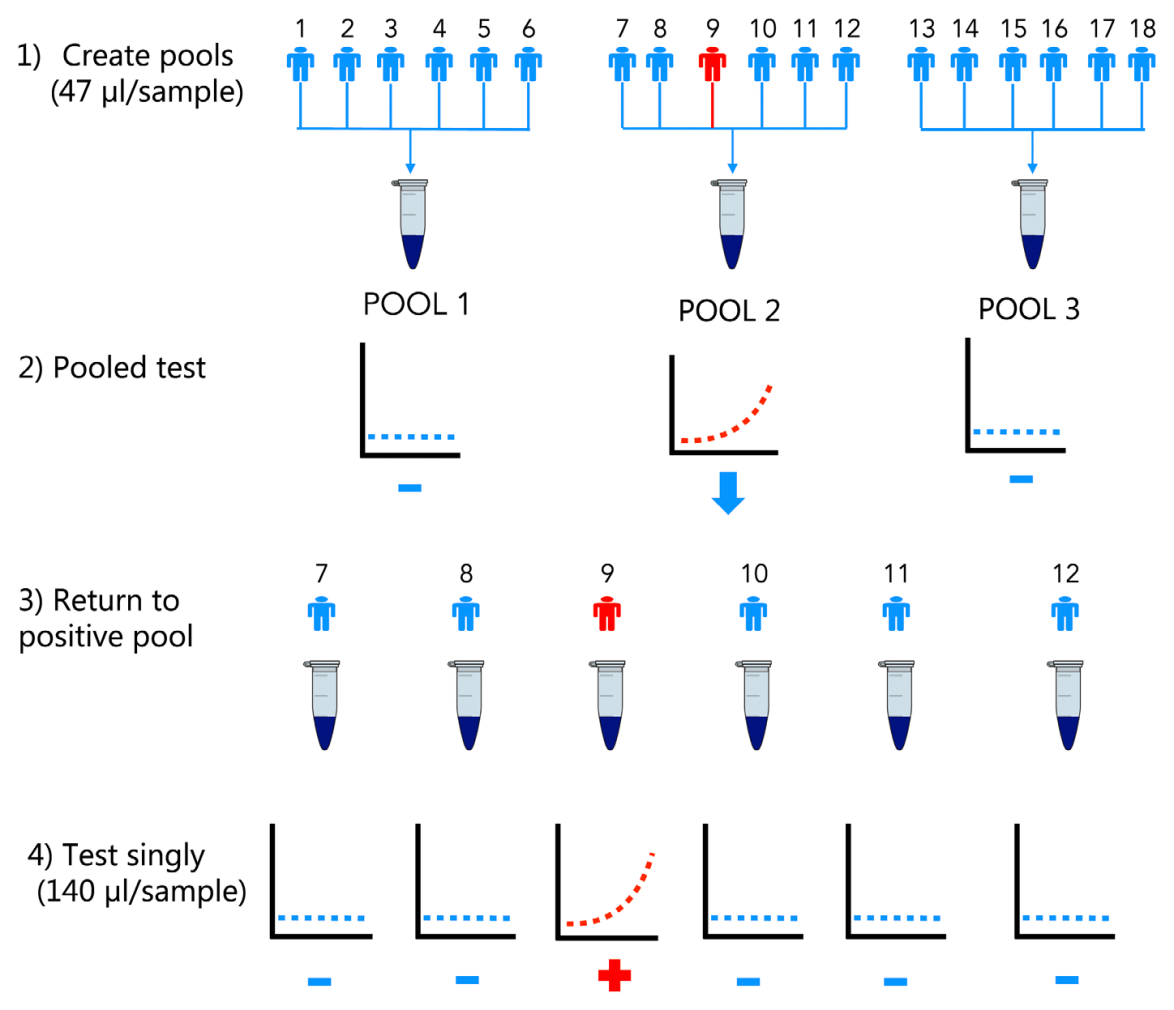
A schema of how the pooled testing strategy works. The example illustrates that if an infection is occurring at about 5.6% (i.e. 1/18) then by the pooled testing, a total of 9 tests can identify the infected individual. In Kenya, the current severe acute respiratory syndrome coronavirus 2 (SARS-CoV-2) positivity rate is ~5.0% thus pooled testing can conserve 50% of testing kits.

Pooled testing is a diagnostic approach where samples from multiple patients are combined and analyzed in a single test reaction ^7^. If the reaction is positive, then individual samples that contributed to that reaction need to be retested singly ( Figure 2). Pooled testing was first used during world war II to efficiently identify syphilis infected military recruits ^8^. More recently, this strategy has been applied in blood banks to screen blood products for HIV-1, hepatitis B and C viruses ^9, 10^. Now, again, this strategy is finding application in identifying SARS-CoV-2 infected individuals in the ongoing COVID-19 pandemic ^7, 11– 15^.

Here, we evaluated whether pooled testing is a viable protocol for SARS-CoV-2 diagnosis in our Kenya setting and potentially other low-to-middle income settings across the globe. We first applied the pooled testing strategy in mid-May 2020 when we were receiving >300 SARS-CoV-2 test requests daily but had access only to the low-throughput manual RNA extraction kits (QIAamp Viral RNA Mini Kits). On applying pooled testing, we were able to keep up with the increasing volume of SARS-CoV-2 test requests daily and have henceforth maintained this strategy even with the high-throughput RNA extraction platforms due to the associated resource conservation.

## Methods

### Study site/location

This study was undertaken at the Kenya Medical Research Institute (KEMRI)-Wellcome Trust Research Programme (KWTRP) located in Kilifi town, on the Kenyan Coast between 21 ^st^ March 2020 and 15 ^th^ July 2020. Since the start of the SARS-CoV-2 epidemic in Kenya, in March 2020, KEMRI-Kilifi has been supporting the County Health Department Rapid Response Teams (RRTs) in coastal region of Kenya in SARS-CoV-2 laboratory testing. Respiratory samples collected by the RRTs for testing are received in 2–3 ml of Universal Transport Media or Virus Transport Media. Data on the aggregated Kenya-wide daily SARS-CoV-2 laboratory tests and number of daily positives were compiled from the Kenya Ministry of Health (MoH) website, specifically the periodic COVID-19 situational reports and the daily press releases.

### Ethics and consent

The work was reviewed internally at KEMRI and considered part of the efforts to promptly develop efficient laboratory protocols for scaling up public health response to the COVID-19 pandemic. As a result, individual patient consent was considered unnecessary for these optimisation experiments. The national daily tally of SARS-CoV-2 tests done and number positive is freely available to the public at the MoH website inclusive of those from KEMRI laboratories.

### Pooled testing protocol

For an optimal pooled testing protocol, there are three key considerations ^16^: (i) the diagnostic protocol limit of detection (LoD) to ensure adequate sample volume is included in the pools, (ii) the diagnostic test sensitivity and specificity and (iii) the prevalence of the infection to guide the optimal pool size (e.g. if infection prevalence reaches 30%, then pooling in groups of 3 would lead to most pools being positive and the need for individual testing, hence no gain in efficiency). In general, pooled testing is most useful when the prevalence of the infection is low (typically <15%) ^17^.

To select the optimal pool size we used the web-based shiny application from Christopher Bilder available at https://www.chrisbilder.com/shiny/ under Hierarchical testing. Assuming a SARS-CoV- 2 prevalence of 4% in our query samples (see later in results section on observed test positivity rate, Figure 1), test sensitivity of 90%, test specificity of 98% ^18^, adoption of a two stage pooling algorithm ( Figure 2) and a pre-specified preferred pool size range of 3-10, the algorithm calculated the optimal testing configuration was a pool size of n=6 followed by individual testing of samples in positive pools.

We excluded from the pooled testing protocol samples collected from known SARS-CoV-2 infected individuals (as per results of our previous test on the same individual) and samples from deceased individuals investigating if they died of COVID-19. This is because the former have a high pre-test probability of being positive thus no need to pool first and for the latter, a fast turn-around time was requested with a definitive result to facilitate decisions around burial rites. Thus, samples from these two groups were processed using a single individual test per sample. For samples processed using the pooled testing protocol, pools were assigned randomly following the consecutive order in which the samples were delivered by the RRTs.

### Laboratory procedures

Our SARS-CoV-2 laboratory testing protocol has been described elsewhere ^19^. Briefly, viral RNA purification from the raw samples was extracted using either of three commercial kits from QIAGEN (Manchester, UK); QIAamp Viral RNA Mini Kit (Catalogue # 52906), RNeasy ^®^ QIAcube ^®^ HT Kit (Catalogue # 74171) and QIASYMPHONY ^®^ RNA Kit (Catalogue # 931636). The manufacturer’s instructions were followed for all the three kits. For the individual samples, viral RNA were extracted from starting volume of 140-µl of raw sample while for the pooled samples viral RNA were extracted from a starting volume of ~280-µl (each sample contributing 47 µl) ( Figure 2). In both cases the purified RNA were collected in 60 µl of elution buffer.

RT-PCR was undertaken using primer/probes from the following four protocols, the details of which we described elsewhere ^19^; (i) the Berlin (Charité) ^20^ (targeting E i.e. envelope gene, N i.e. nucleocapsid gene or RdRp i.e. RNA-dependent RNA-polymerase gene), (ii) European Virus Archive – GLOBAL (EVA-g) (targeting E or RdRp genes), (iii) Da An Gene Co. detection Kit (targeting N or ORF1ab) and Beijing Genomic Institute (BGI) RT-PCR kit (targeting ORF1ab). For the first two protocols only primer/probe mixes from the original protocol were used, as for the other RT-PCR components we used alternative RT-PCR reagents while the latter two are commercial kits that come with all RT-PCR components pre-mixed ready for RT-PCR running after addition of viral RNA extract from patient sample. Only EVA-g E gene protocol that was used in the experiments we described in the results section is further elaborated here in detail.

With the EVA-g assay, 4 µL of the purified RNA (pooled or individual samples) were mixed with 2.5 µl TaqMan ^TM^ Fast Virus 1-Step Master Mix (Applied Biosystems (ABI) Catalogue # 4444436), 1.75 µl E gene primer/ probe mix and 3.75-µl nuclease free water in a real-time PCR plate well. Three controls i.e. run positive control (PC), negative control (NC) and no template control (NTC), were included in every PCR plate for quality assurance and to aid in results interpretation. After sealing and a short spin, the plate was loaded to an ABI 7500 instrument (Thermofisher, USA). The thermocycling conditions used were; 50°C for 5 minutes, then 95°C for 20 seconds followed by 40 cycles of 95°C for 3 second and 58°C for 45 seconds. The amplification curves for all presumptive positive samples were visually inspected prior recording them as confirmed positives. A cycle threshold (Ct) of <38.0 was considered positive for pools and Ct of <37.0 for the individual samples. Lower Ct values indicate more strongly positive samples with more virus quantities.

### Assessment of impact of pooled testing on assay sensitivity

We assessed the impact of pooled testing on test sensitivity by combining a previously identified positive sample (that had been singly analyzed) with five negative samples. We replicated this 6 times. The positive samples were across a range of real-time RT-PCR Ct values (20.65-36.24) ( Table 1 ^21^). The individual positive samples were retested again individually again to compare their repeat test Ct values with their previous test Ct values. 

**Table 1.** Impact of pooled testing on test sensitivity and positive sample results ^¶^.

Sample pool #	Original Ct	Pool Ct	Individual Ct
1 (Pool 1)	20.65	21.88	19.63
2 (Pool 2)	24.78	25.60	23.18
3 (Pool 3)	27.17	30.28	27.17
4 (Pool 4)	29.63	34.18	30.86
5 (Pool 5)	33.36	Negative	35.18
6 (Pool 6)	36.24	Negative	Negative

^¶^Table shows the Ct values obtained when the samples were tested the first time individually (Original Ct), when pooled testing was applied (Pool Ct) and when retested individually again (Individual Ct).

### Data analysis

All numerical data manipulation was undertaken in STATA version 15.1. Positivity rate was calculated by dividing total positives by total samples tested over a specified period with the 95% confidence interval (CI) assuming a binomial distribution. Dispersion of Ct values were summarized using the median and interquartile range (IQR) values. Graphical presentations were generated in R version 3.5.0 using ggplot2 package version 2_3.2.1

## Results

### Optimal pool size and test turnaround

We started SARS-CoV-2 pooled testing on the Kenyan coast on the 14 ^th^ May 2020 ( Figure 1, panel B). The SARS-CoV-2 positivity rate among tested samples in the previous one month period (14 ^th^ April-13 ^th^ May 2020) across Kenya and in our laboratory was ~2.0% (95% CI: 1.8-2.1%) and ~3.3% (95% CI: 2.4-3.8%), respectively. Given the local and national positivity rate among tested samples during this period, and the anticipated increase in the following weeks, we inferred that an n=6 pool size was the optimal at that time point. Note that with similar assumptions of RT-PCR sensitivity and specificity parameters stated in the methods section, for SARS-CoV-2 positivity rates of: 5-7%; 8-15%; and 16-20% a pool size of: n=5, n=4 and n=3 would be recommended, respectively ^6^.

The pooled testing strategy allowed us to screen 471 samples on the first day (14 ^th^ May 2020) of deployment up from 264 the previous day, a 78% increase. Importantly the pooled testing protocol was using QIAamp manual Extraction Mini Kit for viral RNA purification a switch from the high-throughput QIAcube ^®^ HT Kit that we had deployed since 18 ^th^ April 2020 ( Figure 1, panel B).

### Test sensitivity dynamics in pooled testing

The pools that included a strongly positive sample with a Ct value <33.0 also gave a positive result in the pools, while the pools including previously weakly positive samples that had a Ct value above 33.0 gave a negative result in the pools Table 1 ^21^. On repeat testing the previously positive individually, all confirmed positive results except one sample with the previous highest Ct value. This observation is consistent with previous literature on the lack of reproducibility of weak RT-PCR positives especially those close to the test LoD ^22, 23^.

### Example pooled testing result in KEMRI-Kilifi laboratory

**Figure 3.**
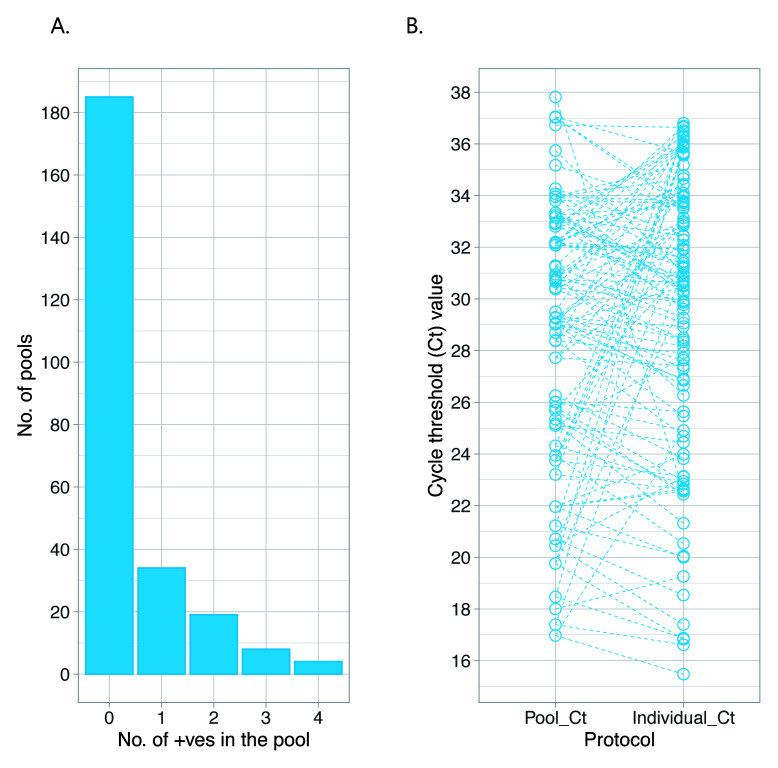
Summary results from the analysis of the 1500 samples analyzed at KEMRI-Kilifi using the pooled testing strategy. Panel **A**, shows the number of positives from the created pools. Panel **B**, comparison of Ct values from pooled tests versus individual sample tests.

To further evaluate the benefit of pooled testing, we examined test results from 1500 samples tested in our laboratory in the first week of June 2020. The clinical records indicated that these samples were from both asymptomatic individuals (n=1009, 72.1%) and symptomatic individuals (n=54, 3.6%). For 364 samples (24.3%), data on the symptom status of the sampled individuals were unavailable. Testing of these samples started with creation of 250 pools (i.e. 6 samples per pool). 75 (30.0%) of the pools gave a positive RT-PCR result. These 75 positive pools were then expanded to 450 individual sample tests and one or more positive samples were identified in 65 pools, a total of 112 positives (i.e. 7.5% of the analysed 1500 samples) ( Figure 3, panel A).

On comparison of the Ct value difference (ΔCt) in the pooled testing and individual sample testing (considering the strongest positive sample only where there were multiple positives in a pool), there was on average a 1.59 Ct value increase during pooled testing when compared to the Ct value of the same samples when tested singly. Expanded pools tested negative in all 6 individual tests in 13.3% of instances (95% CI: 6.6-23.1%), despite a positive result at pool level. The Ct values for expanded pools that were negative for all 6 individual tests ranged 19.73 to 37.83 with median 30.45 (IQR: 26.1-35.66). For the pools where 1 or more positives were identified the Ct values ranged 16.97 to 37.81 with median 30.43 (IQR: 25.68-32.90). False-positive results during pooled testing may arise as a technical artifact of a degraded probe, primer/probe cross-reaction with non-SARS-CoV-2 sequences in some samples or cross-contamination/mislabeling of samples during laboratory processing ^24^.

### Resources conserved in pooled testing

 Overall, in the above example, to get results for 1500 samples we performed 700 tests (RNA extraction and RT-PCR). We estimated that in our laboratory, it costed ~ 6 United States Dollars (USD) per SARS-CoV-2 test. Thus, by undertaking only 46.7% of the tests to identify the positives, using the pooled testing protocol we spent ~ 4200 USD to test the 1500 samples down from ~9000 USD if all samples are tested singly thus saving ~4800 USD. Although two assays were required, because of the overall reduction in numbers of assays, the turnaround time was faster and fewer staff were required to handle the laboratory tests when using the pooled testing approach.

## Conclusions

Pooled testing can yield significant savings of test kits resources while effectively identifying infected SARS-CoV-2 individuals in the population rapidly. This protocol is especially relevant in low-to-middle income settings as testing resources are mostly dependent on limited purchased imports or donations. The strategy further increases test specificity (positives are tested twice) limiting false positives ^25^. However, due to sample dilution, there is a risk of missing weak positives during the first step of pooled testing. The significance of this depends on the reason for testing. For example, if it is to identify who is positive so as to put them into isolation to slow down or stop spread, then there is not much of a loss as weak positives are less likely to contribute to onward transmission. However, if the aim is to identify how many people have been infected to calculate particular epidemiological parameters e.g. infection prevalence, then there is a danger of underestimating these parameters. Strategies that can improve pooled testing protocol sensitivity include (i) increasing the sample volume during nucleic acid extraction (but this has to be balanced with other extraction kit reagents e.g. the lysis buffer) (ii) using high performance nucleic acid extraction kits and (iii) loading a higher volume of extracted nucleic acid from the pooled samples into the RT-PCR reaction. Further, although the overall the sample handling time was reduced, it is difficult to “fast track” individual assays that were declared urgent by clinicians or public health officers where the initial pooled test is positive. Such samples should be excluded from the pooled testing protocol and be processed using the standard single test per sample protocol. As the COVID-19 pandemic evolves, the pool size used by a testing laboratory should be kept under constant review and adjusted if there are changes in the prevalence of the infection in the target population or test accuracy characteristics.

## Data availability

### Underlying data

Replication Data for: Pooled testing conserves SARS-CoV-2 laboratory resources and improves turn-around time: experience at KEMRI-Wellcome Trust Programme, Kenya. https://doi.org/10.7910/DVN/I4XUC5 ^21^

This project contains the following underlying data:

- CAgoti_SARSCoV2_Lab_Experience_Codebook.pdf (Codebook for datasets)
- CAgoti_SARSCOV2_Lab_Experience_Readme.txt (Data description and usage instructions)
- datafiles.zip (Analysis datasets)
- scripts.zip (Analysis scripts)

Data are available under the terms of the Creative Commons Attribution 4.0 International license (CC-BY 4.0).
