# Supplementary material for: Pooled testing conserves SARS-CoV-2 laboratory resources and improves test turn-around time: experience on the Kenyan Coast
Source: Wellcome Open Res. 2021 Feb 3;5:186. Originally published 2020 Aug 6. [Version 2] doi: 10.12688/wellcomeopenres.16113.2 (PMC7590893; doi:10.12688/wellcomeopenres.16113.2)
Supplement: Peer review history

# Peer review: Reviewer response for version 1

**DOI:** 10.21956/wellcomeopenres.17686.r40819

**Published:** 2020-10-30

**Authors:** James A Hay

**Affiliations:** 1 Centre for Communicable Disease Dynamics, Department of Epidemiology, Harvard School of Public Health, Boston, MA, USA

Agoti *et al.* provide a timely and important report on the implementation of pooled SARS-CoV-2 PCR testing on the Kenyan Coast. The manuscript describes the epidemiological context and pooling strategy concisely and clearly. The authors find that pooling drastically increases testing throughput and saves on number of test kits used. Given the hesitancy of many settings to implement pooled testing for fears of logistical challenges and reduced sensitivity, this is an important success story that should be useful to many low-and-middle income settings (and indeed high-income settings!). I really enjoyed reading this paper!

1. The title and results suggest that pooling reduced the test turnaround time. However, I can see no results to support this claim (other than the final, qualitative results sentence). This is a key (potential) benefit of pooling, so please could you provide these results?
2. It would be useful to provide some context on why these tests were carried out. Presumably all of these samples were from a clinical setting, where the considerations for sensitivity (and specificity) may be different than e.g. surveillance or routine screening of HCWs.   
                                    
3. “… by combining a previously identified positive sample … we replicated this 6 times”. This phrasing suggests the same positive sample was diluted 6 different times. Please rephrase to make clear that these were 6 distinct samples across a range of Ct values. Also, were these re-tested samples subjected to an additional freeze-thaw cycle which might degrade RNA?
4. Section “Example pooled testing result in KEMRI-Kilifi laboratory”: “… in our laboratory between in the first week of June 2020”. Typo here (remove between). Also, “The testing started with creation of…”, missing “the”.
5. It would be interesting to discuss where some of the Ct values actually increased from the pooled to individual testing. This could help give an idea of how much testing variation there is in addition to reduced sensitivity through dilution or may simply be due to the presence of both low and high Ct samples in the pool.
6. For the 13.3% of positive pools which had no individually positive samples, was there any retesting or were they assumed false positives? It seems unlikely that all of these pools were false positives (indeed most were likely false negative individual tests). Also, bringing this up here seems a bit at odds with the discussion sentence “The strategy further increases test specificity…”.
7. I think it may be useful to discuss some of the practical lessons learned here. For example, was it easy for staff to adapt their workflow to implement pooling rather than individual testing? How much longer did it take to test a batch using pooling vs. individual testing? I’m sure there are many laboratories around the world that would be interested to hear about the more qualitative lessons learned here.
8. The conclusions are a bit brief. For example, I think readers would like to hear more about the difficulty in fast tracking individual assays. As suggested above, context regarding where and why samples were tested is important, as speed and accuracy requirements may depend on why a test is carried out.
9. As suggested by another reviewer, it would be interesting to see if the number of validation tests required tracked changing % positive.

Is the work clearly and accurately presented and does it cite the current literature?

Yes

If applicable, is the statistical analysis and its interpretation appropriate?

Yes

Are all the source data underlying the results available to ensure full reproducibility?

Yes

Is the study design appropriate and is the work technically sound?

Yes

Are the conclusions drawn adequately supported by the results?

Yes

Are sufficient details of methods and analysis provided to allow replication by others?

Yes

Reviewer Expertise:

Epidemiological modeling. I have focused on strategies to improve and harness PCR testing during the COVID-19 pandemic (including pooling).

I confirm that I have read this submission and believe that I have an appropriate level of expertise to confirm that it is of an acceptable scientific standard.

# Peer review: Reviewer response for version 1

**DOI:** 10.21956/wellcomeopenres.17686.r40816

**Published:** 2020-10-26

**Authors:** Kenny Voon

**Affiliations:** 1 School of Medicine, International Medical University, Kuala Lumpur, Malaysia

The manuscripts is generally well written. Agoti *et al.* describe their experience with SARS-CoV-2 pooled testing in KIMRI-Kilifi. The authors point out that the rational of pooled testing is to conserve resources and had calculated pooling size according to prevalence rate.

 However, several following points in the manuscript should be improved/ clarified.

1. The authors should clarify whether 6- samples-pooled testing were conducted until 16 Jul. 2020.  Based on Figure 1, the percentage of positives seems to have sporadic increase from 21 May until 18 June, which may lead to increase of prevalence.
2. Figure 2B show some pooled CT values are lower than individual Ct values and vise versa. This does not support fully the statement of “RT-PCR  cycle threshold value was -1.59 higher for samples tested in pools compared to samples tested singly’. The authors should clarify why some pooled CT values are lower than individual Ct values.  Were these the effect of several positives in one-pool?
3. Were the pooling being done randomly or by cluster  or stratified?

Is the work clearly and accurately presented and does it cite the current literature?

Yes

If applicable, is the statistical analysis and its interpretation appropriate?

Yes

Are all the source data underlying the results available to ensure full reproducibility?

Partly

Is the study design appropriate and is the work technically sound?

Yes

Are the conclusions drawn adequately supported by the results?

Yes

Are sufficient details of methods and analysis provided to allow replication by others?

Yes

Reviewer Expertise:

Virology, COVID-19 pooling,

I confirm that I have read this submission and believe that I have an appropriate level of expertise to confirm that it is of an acceptable scientific standard.

# Response

**Published:** 2020-10-28

**Authors:** Arnold Wasike Lambisia

**Affiliations:** Kenya Medical Research Institute, Kenya

1. **The authors should clarify whether 6- samples-pooled testing were conducted until 16 Jul. 2020. Based on Figure 1, the percentage of positives seems to have sporadic increase from 21 May until 18 June, which may lead to increase of prevalence. **

*Our response: Yes, the n=6 pool size was maintained from 14 ^th^ May to 31 July 2020 as the high positivity rate spikes during this period were short-lived rather than sustained.*

  

1. **Figure 2B show some pooled CT values are lower than individual Ct values and vice versa. This does not support fully the statement of “RT-PCR cycle threshold value was -1.59 higher for samples tested in pools compared to samples tested singly’. The authors should clarify why some pooled CT values are lower than individual Ct values. Were these the effect of several positives in one-pool? **

*Our response: The reviewer is correct in their interpretation i.e. such was observed in pools with more than one positive. In the result section, we did point this out: “* *On comparison of the Ct value differences (ΔCt) in the pooled testing and individual sample testing ( **considering the strongest positive sample only where there were multiple positives in a pool**), there was on average a 1.59 Ct value increase for the samples during pooled testing versus the same samples tested singly. (* *Note: The reviewer is referring to figure 3B rather than 2B which is relevant to the question)*

  

1. **Were the pooling being done randomly or by cluster or stratified? **

*Our response: Same question was raised by reviewer #1 above (question #3), so same answer.  We have updated this information into the revised version of the manuscript*

# Peer review: Reviewer response for version 1

**DOI:** 10.21956/wellcomeopenres.17686.r39878

**Published:** 2020-08-26

**Authors:** Jesse Gitaka, Ernest Wandera Apondi

**Affiliations:** 1 Kenya Research Station, Institute of Tropical Medicine, KEMRI/Nagasaki University, Nairobi, Kenya; 2 Directorate of Research and Innovation, Mount Kenya University, Thika, Kenya; 3 School of Medicine, Mount Kenya University, Thika, Kenya

Agoti *et al.* share their experience with SARS-CoV-2 pooled testing using real-time reverse transcription polymerase chain reaction (RT-PCR) on the Kenyan Coast. The authors evaluated the viability of pooled testing approach for SARS-CoV-2 diagnosis in low-to-middle income settings such as Kenya. To achieve this, the authors utilized a total of 1500 respiratory samples collected in the coastal region of Kenya during the first week of June 2020. A total of 250 pools (each comprising 6 samples) were subjected to RT-PCR, followed by

 individual testing of samples in positive pools. By employing this strategy, only 700 tests (including RNA extraction and RT-PCR) were conducted to get results for the 1500 samples, translating into a cost reduction of 4,800 USD. Although data analysis reveals increased test specificity, the strategy has an associated risk of reduced sensitivity which might result in false negatives.

 The study has been well conducted and the manuscript well written. The findings of this study are timely in light of the global COVID-19 pandemic that has created an urgent demand for accurate rapid diagnostic strategies to allow for prompt clinical and well-tuned public health interventions. The pandemic has resulted in unprecedented demand on the RT-PCR testing capacity of all countries. Demand for testing has been coupled with a global shortage of commercial kits, reagents, consumables, disruptions in the global transport networks, and exacerbated by international competition for testing resources. Accordingly, even many high-income countries have inadequate RT-PCR testing capacity to effectively suppress ongoing transmission, and most low and middle-income countries (LMICs) are unlikely to be able to establish adequate RT-PCR capacity in the immediate future. Thus, the pooled testing strategy for SARS-CoV-2 offers an attractive solution in molecular testing especially for LMICs.

 However, **minor revisions** are required to accept the manuscript for indexing:

1. The authors described the specificity and sensitivity of the pooled testing strategy for SARS-CoV-2 in general terms. Please provide calculated figures for these measures of test reliability.
2. The authors note in their conclusion that, “due to sample dilution, there is a risk of missing weak positives during the first step of pooled testing.”  It will be useful for the authors to explain the implication of such reduced sensitivity and if there are any mitigation measures that can be employed to help improve the reliability of the test.
3. Was there any method to the pooling of samples? Was it random or non-random?
4. Were the samples from symptomatic or asymptomatic patients?
5. What is the effect of viral loads on pool size and test performance?
6. Please correct a few typographical errors especially under the section, “Example pooled testing result in KEMRI-Kilifi laboratory.”

Is the work clearly and accurately presented and does it cite the current literature?

Yes

If applicable, is the statistical analysis and its interpretation appropriate?

Yes

Are all the source data underlying the results available to ensure full reproducibility?

Yes

Is the study design appropriate and is the work technically sound?

Yes

Are the conclusions drawn adequately supported by the results?

Yes

Are sufficient details of methods and analysis provided to allow replication by others?

Yes

Reviewer Expertise:

Infectious diseases, point-of -care diagnostics development, clinical medicine

We confirm that we have read this submission and believe that we have an appropriate level of expertise to confirm that it is of an acceptable scientific standard.

# Response

**Published:** 2020-10-19

**Authors:** Arnold Wasike Lambisia

**Affiliations:** Kenya Medical Research Institute, Kenya

We thank the handling editor and the peer reviewer for the very important comments and suggestions received. We have revised the manuscript in light of this feedback. Below we provide a point-by-point response to the queries raised by the reviewer. 

 1. The authors described the specificity and sensitivity of the pooled testing strategy for SARS-

 CoV-2 in general terms. Please provide calculated figures for these measures of test reliability.

 We restrained from calculating pooled testing protocol sensitivity and specificity since that required having results on a minimum number of  samples (after sample size calculation) processed in parallel with a gold standard method which we did not have or include in our study. Plans are underway for analyzing a set of samples in our laboratory with the pooled testing protocol and the standard individual testing to provide data on the sensitivity and specificity  of the protocol and this will be presented in our follow up analysis.

  

1. The authors note in their conclusion that, “due to sample dilution, there is a risk of missing weak positives during the first step of pooled testing.” It will be useful for the authors to explain the implication of such reduced sensitivity and if there are any mitigation measures that can be employed to help improve the reliability of the test.

 

 The significance of missing weak positive cases depends on the reason for testing. For example if it is to identify who is positive so as to put them into isolation to slow down or stop spread,  then there is not much loss as weak positives are less likely to contribute to onward transmission. However if the aim is identify how many people have been infected to calculate particular epidemiological parameters e.g. infection prevalence, then there in a danger of their underestimation. Strategies that can improve the sensitivity of the pooled testing protocol include (i) increasing the sample volume during nucleic acid extraction (but this has to be balanced with other  extraction kit reagents e.g. the lysis buffer) (ii) using high performance nucleic acid extraction kits and (iii) loading a higher volume of extracted nucleic acid from the pooled samples into the RT-PCR reaction. We have included this discussion into the revised manuscript.

  

1. Was there any method to the pooling of samples? Was it random or non-random?

Samples collected from a known infected individuals (as per our previous test on the same individual) to check if they were still virus positive and samples from deceased individuals to investigate if they died of COVID-19 were always processed singly. All other samples were processed using the pooled testing protocol. The sample pools were assigned randomly following the consecutive order in which the samples were delivered in the laboratory by the Rapid Response Teams (RRTs).  This information has been clarified in the revised manuscript.

  

1. Were the samples from symptomatic or asymptomatic patients?

The clinical records indicated that these samples were from both asymptomatic individuals (n=1009, 72.1%)  and symptomatic individuals (n=54, 3.6%). For 364 samples (24.3%), information on the symptom status of the sampled individuals was unavailable. We have added this information into the revised manuscript.

  

1. What is the effect of viral loads on pool size and test performance?

When the viral load in a sample is low, a large pool size may lead to a false negative. However, it is not possible to know about the patient viral load before testing. Thus, the pool size  has to be       optimized as described in the paper and elsewhere (considering assay limit of detection, sensitivity, specificity and infection prevalence) to make both economic and  clinical purpose sense.

  

1. Please correct a few typographical errors especially under the section, “Example pooled testing result in KEMRI-Kilifi laboratory.”  

These have been corrected.

 We thank the handling editor and the peer reviewer for the very important comments and suggestions received. We have revised the manuscript in light of this feedback. We have clarified the symptom status of the individuals we tested, the pooled testing protocol inclusion and exclusion criteria and the implications of the sensitivity of pooled testing protocol on application of the arising results. Below we provide a point-by-point response to the queries raised by the reviewer. 

 1. The authors described the specificity and sensitivity of the pooled testing strategy for SARS-

 CoV-2 in general terms. Please provide calculated figures for these measures of test reliability.

 We restrained from calculating pooled testing protocol sensitivity and specificity since that required having results on a minimum number of  samples (after sample size calculation) processed in parallel with a gold standard method which we did not have or include in our study. Plans are underway for analyzing a set of samples in our laboratory with the pooled testing protocol and the standard individual testing to provide data on the sensitivity and specificity  of the protocol and this will be presented in our follow up analysis.

  

1. The authors note in their conclusion that, “due to sample dilution, there is a risk of missing weak positives during the first step of pooled testing.” It will be useful for the authors to explain the implication of such reduced sensitivity and if there are any mitigation measures that can be employed to help improve the reliability of the test.

 

 The significance of missing weak positive cases depends on the reason for testing. For example if it is to identify who is positive so as to put them into isolation to slow down or stop spread,  then there is not much loss as weak positives are less likely to contribute to onward transmission. However if the aim is identify how many people have been infected to calculate particular epidemiological parameters e.g. infection prevalence, then there in a danger of their underestimation. Strategies that can improve the sensitivity of the pooled testing protocol include (i) increasing the sample volume during nucleic acid extraction (but this has to be balanced with other  extraction kit reagents e.g. the lysis buffer) (ii) using high performance nucleic acid extraction kits and (iii) loading a higher volume of extracted nucleic acid from the pooled samples into the RT-PCR reaction. We have included this discussion into the revised manuscript.

  

1. Was there any method to the pooling of samples? Was it random or non-random?

Samples collected from a known infected individuals (as per our previous test on the same individual) to check if they were still virus positive and samples from deceased individuals to investigate if they died of COVID-19 were always processed singly. All other samples were processed using the pooled testing protocol. The sample pools were assigned randomly following the consecutive order in which the samples were delivered in the laboratory by the Rapid Response Teams (RRTs).  This information has been clarified in the revised manuscript.

  

1. Were the samples from symptomatic or asymptomatic patients?

The clinical records indicated that these samples were from both asymptomatic individuals (n=1009, 72.1%)  and symptomatic individuals (n=54, 3.6%). For 364 samples (24.3%), information on the symptom status of the sampled individuals was unavailable. We have added this information into the revised manuscript.

  

1. What is the effect of viral loads on pool size and test performance?

When the viral load in a sample is low, a large pool size may lead to a false negative. However, it is not possible to know about the patient viral load before testing. Thus, the pool size  has to be       optimized as described in the paper and elsewhere (considering assay limit of detection, sensitivity, specificity and infection prevalence) to make both economic and  clinical purpose sense.

  

1. Please correct a few typographical errors especially under the section, “Example pooled testing result in KEMRI-Kilifi laboratory.”  

These have been corrected.
